# miRNA-874-3p inhibits the migration, invasion and proliferation of breast cancer cells by targeting VDAC1

**DOI:** 10.18632/aging.204474

**Published:** 2023-01-10

**Authors:** Housheng Yang, Zhiwen Wang, Shuang Hu, Lu Chen, Wei Li, Zhongyi Yang

**Affiliations:** 1 School of Medicine, Hunan Normal University, Changsha 414006, Hunan, P.R. China; 2 Key Laboratory of Chronic Noncommunicable Diseases, Yueyang Vocational Technical College, Yueyang 414006, Hunan, P.R. China; 3 Yueyang Engineering Technology Research Center of Breast Disease Diagnosis and Treatment, Yueyang People’s Hospital, Yueyang Hospital Affiliated to Hunan Normal University, Yueyang 414006, Hunan, P.R. China; 4 College of Health, Dongguan Polytechnic, Dongguan 523808, Guangdong, P.R. China

**Keywords:** breast cancer, VDAC1, miRNA-874-3p

## Abstract

Breast cancer is an important cause of crisis for women’s life and health. Voltage-dependent anion channel 1 (VDAC1) is mainly localized in the outer mitochondrial membrane of all eukaryotes, and it plays a crucial role in the cell as the main interface between mitochondria and cellular metabolism. Through bioinformatics, we found that VDAC1 is abnormally highly expressed in breast cancer, and the prognosis of breast cancer patients with high VDAC1 expression is poor. Through *in vivo* and *in vitro* experiments, we found that VDAC1 can promote the proliferation, migration and invasion of breast cancer cells. Further research we found that VDAC1 can activate the wnt signaling pathway. Through analysis, we found that miR-874-3p can regulate the expression of VDAC1, and the expression of miR-874-3p is decreased in breast cancer, resulting in the increase of VDAC1 expression. Our findings will provide new targets and ideas for the prevention and treatment of breast cancer.

## INTRODUCTION

As the most prevalent malignant solid tumor in the world, breast cancer has a devastating impact on the lives of women [1, 2]. With the advancement of medical technology and the rising popularity of early breast cancer screening, the 5-year survival rate for early breast cancer patients after surgery, radiotherapy, and chemotherapy can reach 90 percent. However, the late breast tumor by direct infiltration or with the blood and lymph flow, transfer to other tissues or organs, and growth result in a poor prognosis for advanced breast cancer patients, with a 5-year survival rate of only about 26%, which has a significant impact on patients’ survival time and quality [3–5]. Moreover, due to the lack of early tumor-specific indicators or negligible initial symptoms of breast cancer, patients are diagnosed at stage III or IV and even miss the optimal treatment period, resulting in varied degrees of physical and mental burden [6, 7]. The molecular mechanism behind the onset and progression of breast cancer is still unclear. Consequently, research into the pathogenesis is critical for the development of effective therapies for breast cancer.

miRNA is an endogenous single-stranded non-coding RNA molecule composed of more than 20 nucleotides [8]. MiRNA mainly plays the role of gene regulation at the post-transcriptional level, and plays the role of tumor suppressor gene or oncogene by targeting mRNA degradation or inhibiting its translation process [9]. MiRNAs can affect the molecular expression of tumor cells through a variety of ways, thus affecting the ability of cell adhesion, changing the cytoskeleton structure, and participating in the regulation of cell-extracellular matrix interaction [10, 11]. Some scholars have demonstrated that miRNA can affect tumor bioactivity by regulating the signaling pathway of target genes. Cancer-related miRNAs are usually divided into two types. The first type of carcinogenic miRNA is often highly expressed in tumors, where it can promote the incidence and development of tumors and play a crucial role in tumor phenotypic maintenance. By controlling cell proliferation, apoptosis, and other processes, the second class of tumor suppressor microRNA decreases the occurrence, development, and medication resistance of cancers. In malignant tumors, miRNAs can therefore play either synergistic or antagonistic roles [12, 13]. It has been reported that miR-100, miR-122, miR-145, and miR-205 are down-regulated in breast cancer [14–17], while miR-1228, miR-150, miR-155, and miR-330-3p are up-regulated in breast cancer [18–21]. Recent study has shown that miR-874-3p expression is aberrant in liver cancer, osteosarcoma, colon cancer, and ovarian cancer [22–25], but our data shows that the expression of miR-874-3p is lowered in breast cancer.

Voltage-dependent anion channel 1 (VDAC1) is primarily positioned in the outer mitochondrial membrane of all eukaryotes and is the fundamental link between mitochondria and cellular metabolism [26]. VDAC1 has been demonstrated to be related with a number of disorders in the current investigation, and it is significantly expressed in a variety of tumors [27]. VDAC1 supports their metabolism via the transfer of diverse metabolites and mitochondrial ATP binding, resulting in mitochondrial regulation of glycolytic flow through the TCA cycle and the action of ATP synthase to meet tumor demand for metabolites or metabolite precursors [28]. By interacting with the anti-apoptotic proteins Bcl-XL, Bcl-2, and HK, VDAC1 regulates cancer cell apoptosis and shields tumor cells from cell death [29]. In addition, hyperglycemia increases VDAC1 expression in pancreatic -cells and kidneys, and VDAC1 levels are elevated in mouse coronary endothelial cells isolated from diabetic mice, because glucose-stimulated insulin secretion is dependent on the production of ATP and other metabolites in mitochondria, and VDAC1 regulates energy and metabolism [30]. Consequently, VDAC1 is necessary for insulin secretion.

We detected miR-874-3p and VDAC1 expression in breast cancer cells and tissues in our investigation. The targeted regulatory link between miR-874-3p and VDAC1 was confirmed, along with the associations between miR-874-3p expression, patient clinicopathology, and prognosis.

## RESULTS

### VDAC1 expression is elevated in breast cancer

**Figure 1:**
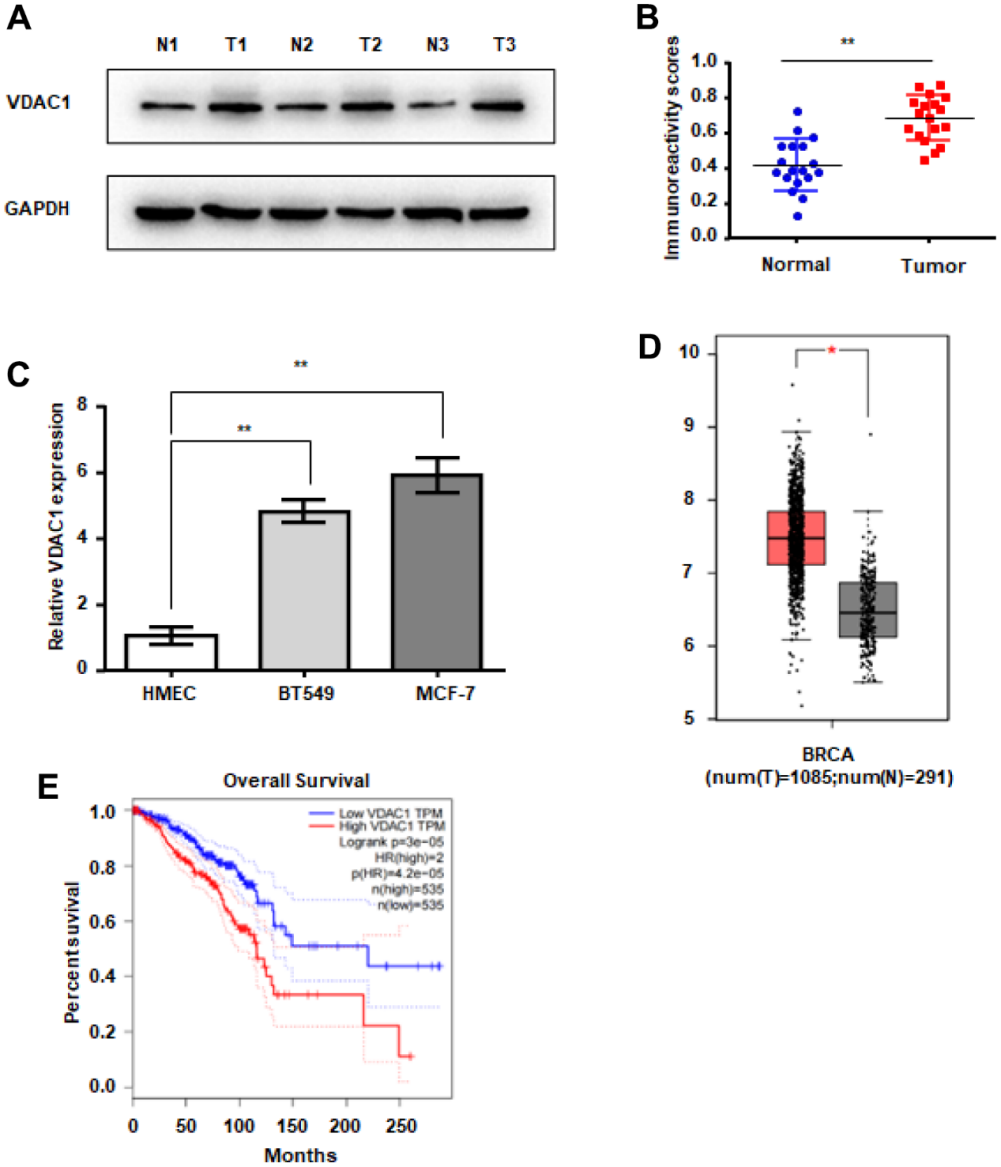
**VDAC1 is highly expressed in breast cancer.** (**A**, **B**) Analysis of VDAC1 expression in 18 breast cancer tissues and adjacent paired normal breast tissue samples. Representative western blotting images of VDAC1 levels in three breast cancer tissues and three normal breast tissues (**A**). VDAC1 and GAPDH protein levels were determined using ImageJ (**B**). (**C**) RT-qPCR was used to detect the mRNA expression of VDAC1 in normal breast cells and breast cancer cells. (**D**) Expression of VDAC1 in normal tissues and breast cancer tissues from the TCGA database. (**E**) Kaplan-Meier survival analysis of breast patients with positive or negative VDAC1 expression. The data are presented as the mean ± S.D. *P < 0.05, **P < 0.01.

To determine the expression of VDAC1 in human breast cancer, western blot was utilized to identify the expression of VDAC1 in human breast cancer and normal breast tissues. Western blotting revealed that VDAC1 protein expression was considerably higher in breast cancer tissues than in surrounding tissues (Figure 1A, 1B). Simultaneously, we examined the expression of VDAC1 in human breast cancer cells and human mammary epithelial cells, and the findings revealed that, compared with normal mammary epithelial cells HMEC, the expression of VDAC1 was higher in human breast cancer cells BT549 and MCF-7 (Figure 1C). By studying the TCGA database, we discovered that the expression of VDAC1 is elevated in breast cancer patients, and that these patients had a bad prognosis (Figure 1D, 1E). All of these results indicate that VDAC1 may be associated with the incidence of breast cancer.

### VDAC1 promotes the proliferation, migration and invasion of breast cancer cells

**Figure 2:**
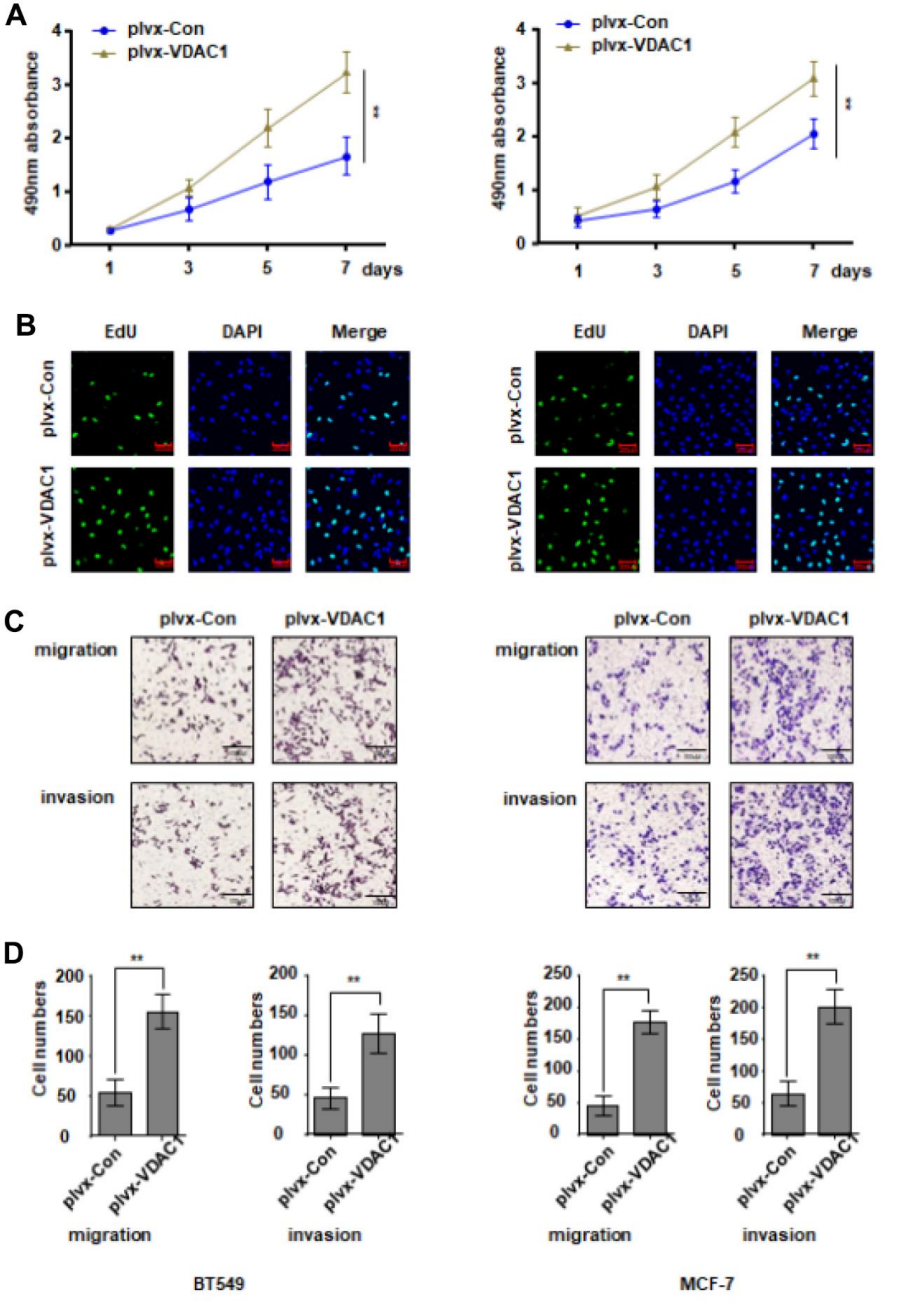
**VDAC1 promotes the proliferation, migration and invasion of breast cancer.** The VDAC1 overexpression plasmid plvx-VDAC1 or blank plasmid plvx-Con was transfected into BT549 and MCF-7 cells. (**A**, **B**) Cell proliferation was detected with CCK8 assay (**A**) and EDU assay (**B**). (**C**, **D**) Transwell assay was used to detect cell migration and invasion ability. The data are presented as the mean ± S.D. *P < 0.05, **P < 0.01.

Next, we evaluated the impact of VDAC1 on breast cancer *in vitro* by transfecting BT549 and McF-7 cells with VDAC1 overexpression plasmid plvx-VDAC1 and control plasmid plvx-Con, respectively. Overexpression of VDAC1 significantly boosted the proliferative potential of breast cancer cells compared to the control group, as determined by CCK8 (Figure 2A). In addition, EdU measurements revealed that in the VDAC1 overexpression group, the fraction of cells in the replication phase was considerably elevated (Figure 2B). The findings of the Transwell experiment indicated further that VDAC1 overexpression enhances the capacity of breast cancer cells to move and invade (Figure 2C, 2D). All of these data demonstrated that VDAC1 can enhance breast cancer malignancy *in vitro*.

### VDAC1 promotes the occurrence and development of breast cancer *in vivo*

**Figure 3:**
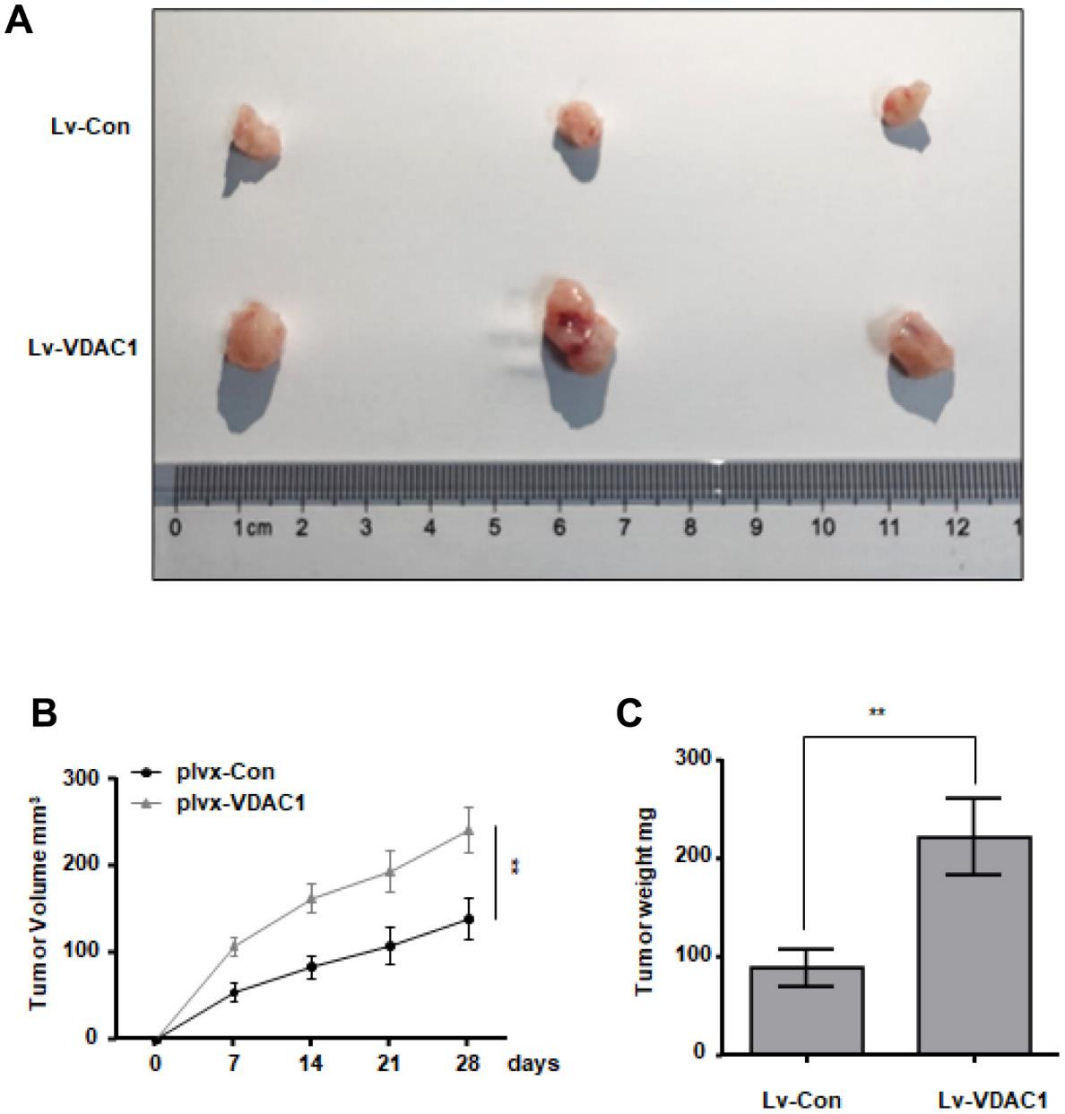
**VDAC1 promotes breast cancer growth *in vivo*.** Subcutaneous xenografts of MCF7 cells infected with VDAC1 overexpressing lentivirus or control lentivirus. (**A**) Images of the tumors at autopsy from nude mice are presented. (**B**, **C**) Tumor volumes (**B**) and average weight (**C**) of xenografted tumors were measured. Data represent the means ± S.D. **P < 0.01.

To investigate the influence of VDAC1 on breast cancer *in vivo*, MCF-7 cells stably overexpressed with VDAC1 and control cells were subcutaneously injected into immunodeficient mice to provide a subcutaneous tumor-bearing model of human breast cancer. The observation was started on the seventh day. Every four days, tumor growth and size were recorded. After 28 days, mice were killed and tumors were removed. The tumors generated in the VDAC1 overexpression group (plvx-VDAC1) were considerably larger than those in the control group (plvx-Con), as measured by tumor volume (Figure 3A–3C). *In vitro* and *in vivo* studies demonstrate that VDAC1 promotes breast cancer.

### miR-874-3p directly binds to the VDAC1 3’-UTR

**Figure 4:**
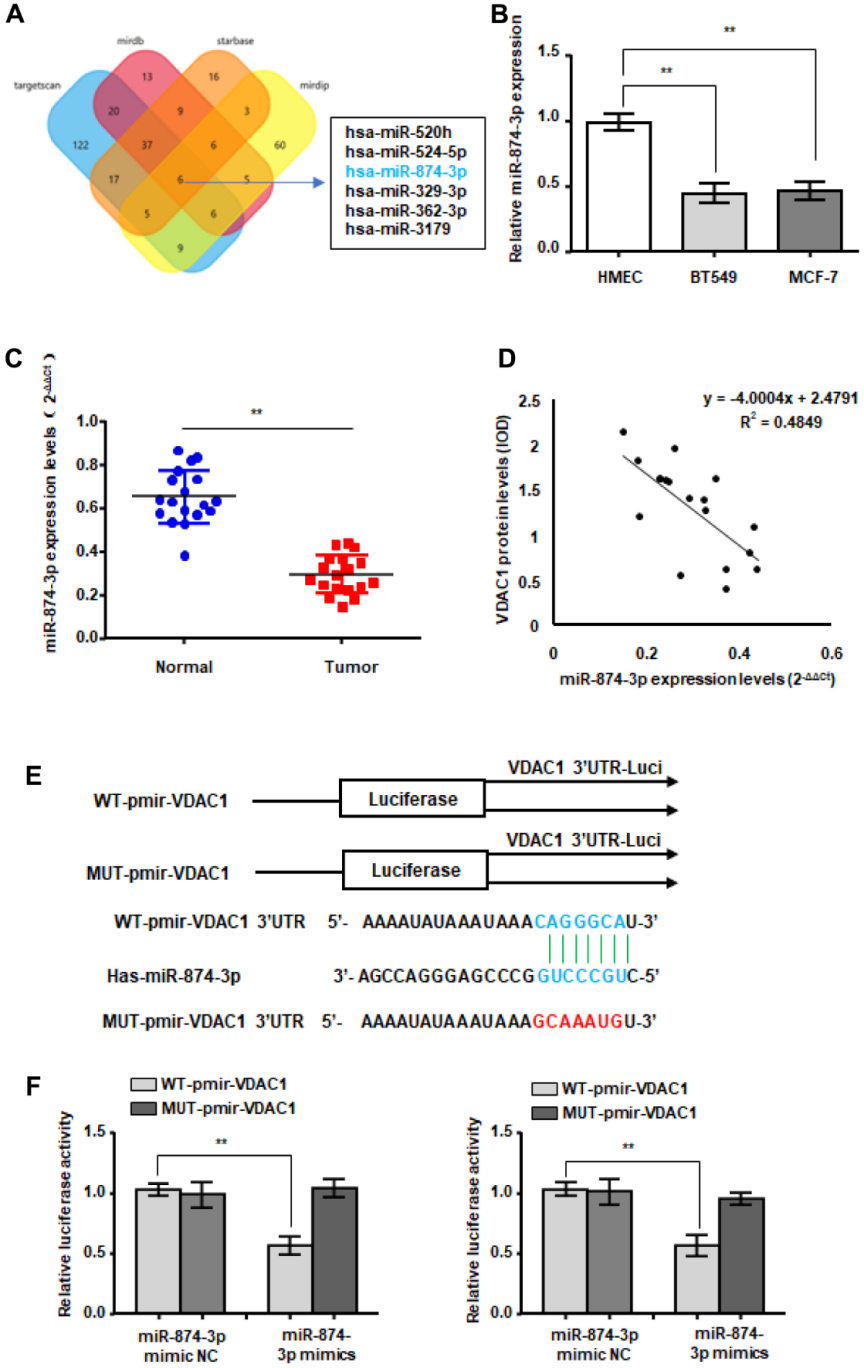
**WTX is a direct target of miR-874-3p.** (**A**) The four-way Venn diagram reveals the numbers of overlapping miRNAs obtained using four publicly available bioinformatics algorithms and the microarray-based VDAC1 signature. (**B**) RT-qPCR was used to detect the relative expression of miR-874-3p in normal breast cells and breast cancer cells. (**C**) RT-qPCR was used to detect the expression in 18 breast cancer tissues and adjacent paired normal breast tissue samples. (**D**) Correlation between miR-874-3p levels and VDAC1 levels in 18 breast cancer tissues. (**E**) Nucleotide predicted miR-874-3p-binding site in the VDAC1 mRNA 3′-UTR. (**F**) MCF-7 and BT549 cells were transfected with reporter plasmids containing WT-pmir-VDAC1 or MUT-pmir-VDAC1 and miR-874-3p mimic or miR-874-3p mimic NC, and luciferase activity was detected. Data represent the means ± S.D. **P < 0.01.

To explore the reasons for the abnormally high expression of VDAC1 in breast cancer, we predicted the miRNAs that bind to the 3’UTR region of VDAC1 mRNA through a bioinformatics website (Figure 4A). In accordance with our predictions, RT-qPCR detection revealed that only miR-874-3p was decreased in breast cancer (Figure 4B). Additionally, we discovered that miR-874-3p was downregulated in breast cancer tissues and was negatively linked with the expression of VDAC1 (Figure 4C, 4D). To establish the targeting interaction between miR-874-3p and VDAC1, luciferase reporter-tagged wild-type and mutant plasmids were generated (Figure 4E). The findings demonstrated that miR-874-3p mimics had no effect on the luciferase activity of mutant plasmids in breast cancer cells, but reduced the luciferase activity of wild-type plasmids (Figure 4F).

### miR-874-3p inhibits the malignancy of breast cancer cells

**Figure 5:**
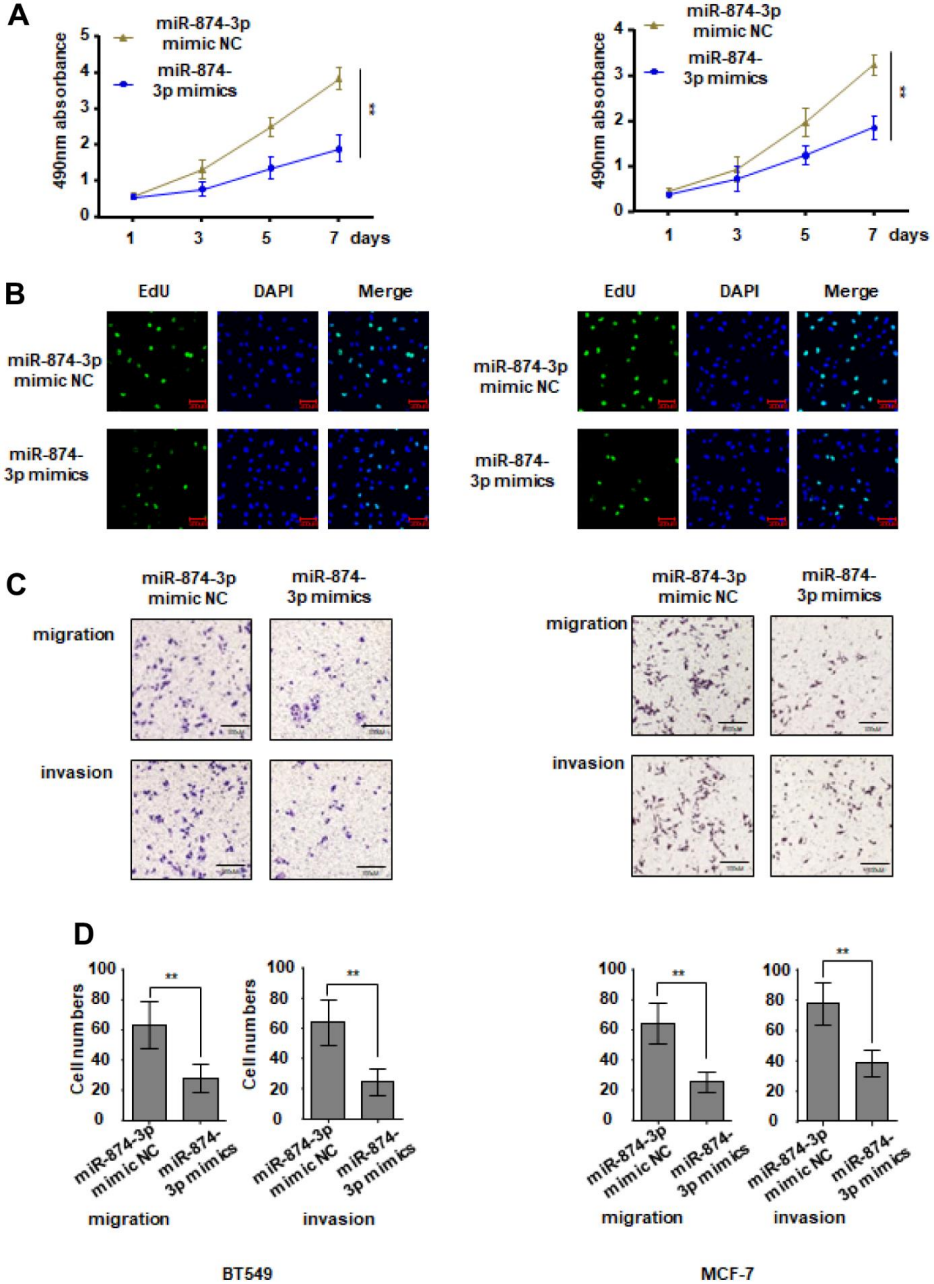
**miR-874-3p inhibits proliferation, migration and invasion of breast cancer cells.** MCF-7 and BT549 cells were transduced with miR-874-3p mimic NC or miR-874-3p mimics. (**A**, **B**) Cell proliferation was detected with CCK8 assay (**A**) and EDU assay (**B**). (**C**, **D**) Transwell assay was used to detect cell migration and invasion ability. The data are presented as the mean ± S.D. *P < 0.05, **P < 0.01.

During this period, breast cancer cells were transfected with miR-874-3p mimics or controls without mimics. Compared with the control group, the proliferation ability of MCF-7 and BT549 cells transfected with miR-874-3p mimic was significantly inhibited (Figure 5A, 5B). The Transwell experiment showed that transfection of miR-874-3p mimics made BT549 and MCF-7 cells less likely to move and invade compared to the blank control (Figure 5C, 5D).

### miR-874-3p-VDAC1 axis regulates Wnt/β-catenin signaling in breast cells

**Figure 6:**
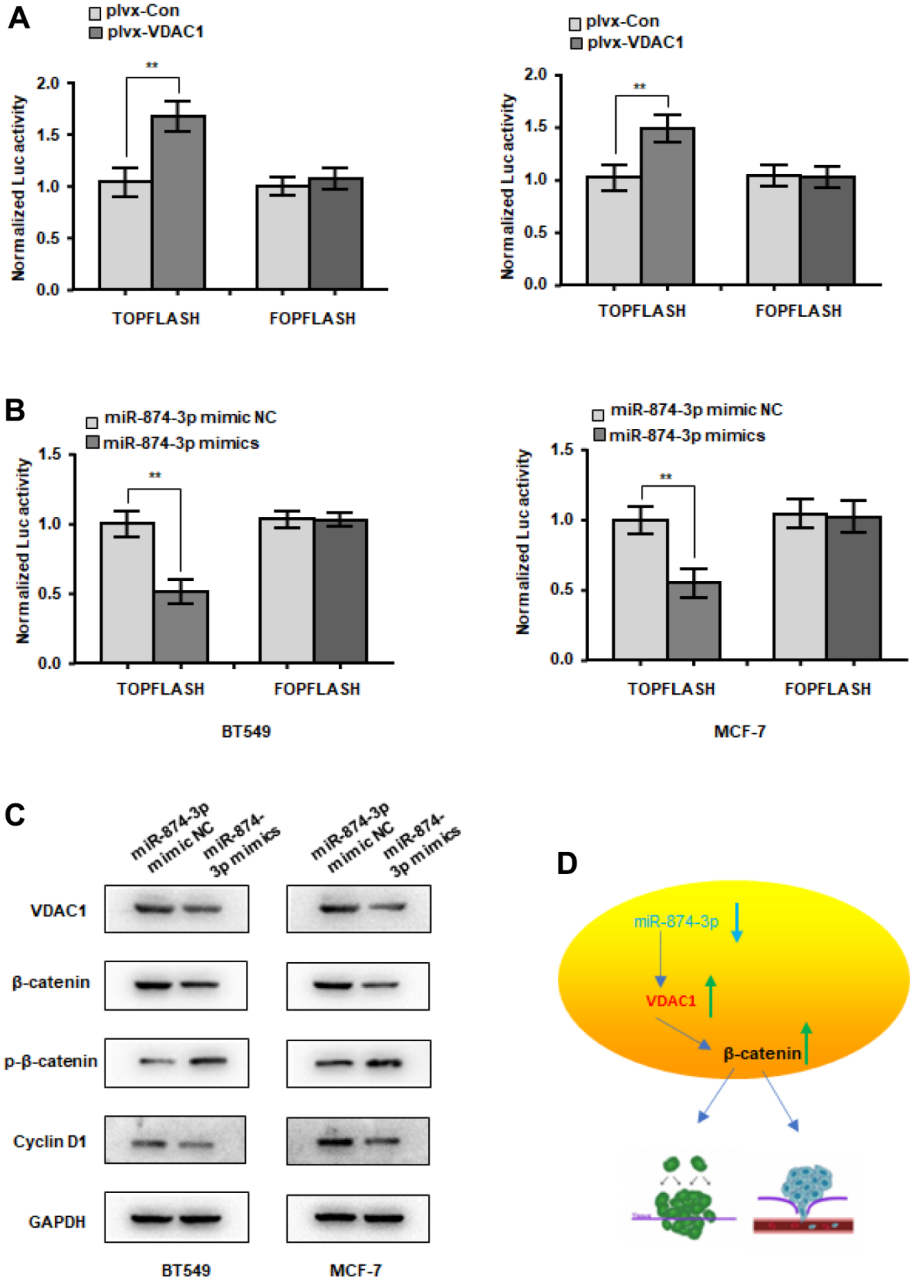
**miR-874-3p-VDAC1 axis regulates Wnt/β-catenin signaling in breast cancer cells.** (**A**) β-catenin reporter assay in MCF-7 and BT549 cells with VDAC1 overexpression (**A**) or miR-874-3p overexpression (**B**). (**C**) Effects of miR-874-3p on protein levels of total β-catenin, phosphorylated β-catenin (Ser33/37/Thr41) and cyclin D1. (**D**) Schematic of the mechanism. The data are presented as the mean ± S.D. *P < 0.05, **P < 0.01.

The Wnt signaling pathway is a classic signaling pathway in tumors. Abnormal activation of wnt signaling often leads to tumorigenesis. Will VDAC1 activate wnt signaling? In MCF-7 and BT549 cells, VDAC1 can increase the activity of the β-catenin reporter gene (Figure 6A), although miR-874-3p has the opposite effect (Figure 6B). In addition, we discovered that the expression of β-catenin and downstream Cyclin D1 was reduced, while the expression of p-β-catenin was elevated, following transfection with miR-874-3p mimics (Figure 6C). These findings suggest that VDAC1 enhances the onset and progression of breast cancer via stimulating the wnt signaling pathway.

## DISCUSSION

Breast cancer is a common malignant tumor, accounting for 20% of female malignant tumors, the latest research reports show that breast cancer has become the highest incidence of cancer in the world, seriously affecting the survival of patients [31]. Our previous study discovered a link between VDAC1 and invasion and metastasis of breast cancer cell proliferation, but VDAC1 in the molecular regulation mechanism of breast cancer is unclear, so the VDAC1 in the incidence of breast cancer, development of molecular mechanism, and study the related regulation can help to find new breast cancer treatment targets, and promote the development of precision medical treatment of breast cancer. This gene has been discovered to be overexpressed in numerous malignancies, including lung cancer, cervical cancer, gastric cancer, pancreatic cancer, and laryngeal cancer [32–36]. In 2012, Brahimi-Horn et al., in their research on the drug resistance of lung cancer, found that cell lines with high expression of VDAC1 gene had higher resistance to apoptosis induced by staurosporine and etoposide, and silenced VDAC1 gene. Afterwards, the cell line regained its sensitivity to the above-mentioned drugs. In addition, high expression levels of VDAC1 gene can be detected in advanced lung cancer and tissues with larger lung cancer. It is concluded that VDAC1 may become a novel marker for early diagnosis and prognosis evaluation of lung cancer [37]. In 2010, Lan et al. found in their study on the mechanism of apoptosis in gastric cancer that the up-regulation of complexes and the down-regulation of proteins play a key role in the process of mediated apoptosis [34]. These results suggest that it is necessary to explore the role of VDAC1 in cancer.

Non-coding RNAs (ncRNAs) are RNA molecules that are produced by transcription but do not code for proteins; these molecules mostly consist of LncRNAs, CircRNAs, miRNAs, etc. At the post-transcriptional level, they play a significant role in gene expression regulation and epigenetic regulation [38]. This research investigated the function of microRNAs in the onset and progression of breast cancer. miRNAs are directly associated to the advancement of breast cancer, and they play varied roles at different phases of breast cancer development due to their involvement in numerous cancer processes. We believe miRNAs are involved in the regulation of malignant proliferation, escape from growth inhibition, cellular senescence, and genomic instability, despite the fact that their functions in numerous cancer processes have not been demonstrated. Several microRNAs have been discovered to be inappropriately expressed in breast tumors during the earliest stages of malignancy. VDAC1 is a miR-874-3p target gene, and miR-874-3p inhibits its expression in breast cancer cells. miR-874-3p has been shown to affect the expression of a variety of target genes, including GDPD5, FOXM1, FAM84A, and ADAM19 [22, 24, 39, 40]. However, no articles have reported the targeting link between miR-874-3p and VDAC1 and the role of miR-874-3p in breast cancer.

According to this study, miR-874-3p suppresses the proliferation, migration, and invasion of breast cancer cells as well as the expression of VDAC1, regulates the cell cycle, and promotes the death of breast cancer cells, as shown in Figure 6D. Therefore, miR-874-3p may be used as a biomarker for the early diagnosis of breast cancer. VDAC1 was validated as a target gene of miR-874-3p in the breast cancer molecular regulation mechanism, and its molecular regulation mechanism was investigated. We will improve the molecular regulatory mechanism of VDAC1 in the future. The findings imply that miR-874-3p may also serve as a novel therapeutic target for breast cancer, shedding fresh light on the investigation of miRNA expression profiles in breast cancer.

## MATERIALS AND METHODS

### Tissue specimens

The breast cancer tissue specimens used in this study were obtained from patients who underwent breast cancer resection in Yueyang People’s Hospital.

### Cell lines and cell culture

The BT549 and MCF-7 breast cancer cell lines were obtained from the Shanghai Cell Bank of the Chinese Academy of Sciences (Shanghai, China). MCF-7 was grown in DMEM medium (BI, Israel) with 10% fetal bovine serum, whereas BT549 was grown in RPMI 1640 medium (BI, Israel) with 10% fetal bovine serum. All cells were grown at 37° C in an incubator containing 5% CO_2_.

To construct cell lines stably overexpressing VDAC1, we infected MCF-7 cells with lentivirus containing VDAC1 and used 500 ng/mL puromycin for resistance selection. Cells were harvested a week later for verification.

### qRT-PCR

Firstly, Trizol was added to the collected cells or tissues for mixing, then a quarter volume of chloroform was added, and then the supernatant was centrifuged, and the same volume of isopropanol was added for RNA deposition, all at 4° C. 1ugRNA was added to gDNA Wiper Mix solution to remove genomic DNA, and then HiScript qRT superMix solution was added for reverse transcription to obtain cDNA. Then take 2 μL of cDNA and add qPCR SuperMix solution for qRT-PCR. Primer sequences are as follows: VDAC1 forward, 5′-GGTGCTCTGGTGCTAGGTTA-3′ and reverse, 5′-CAGCGGTCTCCAACTTCTTG-3′ and GAPDH forward, 5′-ACCCACTCCTCCACCTTTGAC-3′ and reverse, 5′-TGTTGCTGTAGCCAAATTCGTT-3′.

### Plasmids, miRNAs, and transfection

The plvx-VDAC1 plasmid and miR-874-3p mimics were obtained from Origene (Beijing, China). miR-874-3p mimics sequence: 5′-ACUGCGUUGAAACAUGGGUA-3′, and the sequence of the miR-mimic NC was 5′-UUGAGGCUUCAAUCGACGUTT-3′. All transfections were carried out utilizing Lipo2000 per the directions. First, cells were seeded in 6-well plates, then transfected 24 hours later, followed by additional functional testing 24 hours following transfection.

### Transwell invasion and migration assays

36 hours after transfection, 5 × 10^4^ cells were planted to the transwell chamber, the cells were incubated in the incubator for 36 hours, washed with PBS solution, cleaned with sterile cotton swabs, fixed with methanol, stained with crystal violet, and dried in a ventilation cabinet. Under an inverted microscope, photographs were taken and numbered. In preparation of the cell invasion assay, matrigel was spread in the Transwell chamber and dried overnight.

### CCK8 assay

Transfected cells were inoculated into 96-well plates and absorbance was measured at 0, 1, 3, 5 and 7 days, respectively. Before each measurement, 10 μL CCK8 (Sigma) solution was added to each well and incubated for 2 h. At a wavelength of 450 nm, the absorbance of each well was measured.

### EdU assay

The transfected cells were counted and then seeded on cell slides, EdU working solution was added at 24 hours and incubated for 3 hours, fixed with paraformaldehyde, and then EdU reaction solution was added, and photographed and analyzed under a fluorescence microscope.

### Western blotting

The cells or tissues were treated with RIPA lysate, properly mixed, centrifuged, and the supernatant was added to electrophoresis loading buffer. The separated proteins in the gel were transferred to PVDF membranes, coated with primary antibody overnight, incubated with secondary antibody the next day, and then exposed and developed using a gel imaging system. Primary antibodies used were anti-VDAC1 (A19707; Abclonal), anti-β-Catenin (A0316; Abclonal), phosphorylated β-catenin (AP1076; Abclonal), anti-Cyclin D1 (A0310; Abclonal), anti-GAPDH (A19056; Abclonal).

### Animal studies

In this study, 10 Balb/ C female nude mice (4 weeks old) were purchased from Beijing Spaifu Company and kept under SPF (temperature 25° C, humidity 55%). The Yueyang People’s Hospital Animal Ethics Committee authorized the trial. Mice were randomly assigned into two groups corresponding to the MCF-7-control group and MCF-7-overexpression group, and then the cells in different treatment groups were mixed with matrix glue at 1:1 and adjusted to a cell suspension of 5×10^7^/ mL cells. Mice were subcutaneously sown with cells using a standard 1 ml syringe (100μl, 5×10^6^ cells per mouse).

### Statistical analysis

This study repeated each experiment at least three times. GraphPad Prism 8.0 was utilized to process and assess the experimental data. The data are given as the standard deviation of the mean. Student’s t-test, chi-square test, and Kaplan Meier analysis were utilized in the statistical study. P<0.05 signifies that the outcomes of the experiment are statistically significant.
